# An Overview of Epithelial-to-Mesenchymal Transition and Mesenchymal-to-Epithelial Transition in Canine Tumors: How Far Have We Come?

**DOI:** 10.3390/vetsci10010019

**Published:** 2022-12-28

**Authors:** Federico Armando, Federico Mazzola, Luca Ferrari, Attilio Corradi

**Affiliations:** 1 Department of Pathology, University of Veterinary Medicine, Foundation, 30559 Hannover, Germany; 2 Department of Veterinary Science, University of Parma, 43100 Parma, Italy

**Keywords:** epithelial-to-mesenchymal transition (EMT), mesenchymal-to-epithelial transition (MET), canine tumors, translational medicine

## Abstract

**Simple Summary:**

This review deals with the general notion of EMT and the main factors regulating this process, including transcription factors, microRNAs, reactive oxygen species, exosomes, microvesicles, and viruses. Articles dealing with EMT in embryonic life, EMT in fibrosis, as well as in cancer metastasis are discussed, including those dealing with EMT and the tumor microenvironment, pre-metastatic niches, and cancer stem cells. Subsequently, a more in depth analysis of articles reporting EMT in relevant epithelial cancers such as mammary gland carcinomas, prostatic carcinomas, and others is provided. Articles that reported results on the use of EMT as a prognostic marker are also selected and discussed. In addition, the general notion of MET and, in more detail, of MET in sarcomas is discussed. Finally, the use of therapeutic approaches for EMT and MET is addressed.

**Abstract:**

Historically, pre-clinical and clinical studies in human medicine have provided new insights, pushing forward the contemporary knowledge. The new results represented a motivation for investigators in specific fields of veterinary medicine, who addressed the same research topics from different perspectives in studies based on experimental and spontaneous animal disease models. The study of different pheno-genotypic contexts contributes to the confirmation of translational models of pathologic mechanisms. This review provides an overview of EMT and MET processes in both human and canine species. While human medicine rapidly advances, having a large amount of information available, veterinary medicine is not at the same level. This situation should provide motivation for the veterinary medicine research field, to apply the knowledge on humans to research in pets. By merging the knowledge of these two disciplines, better and faster results can be achieved, thus improving human and canine health.

## 1. Introduction

Cancer in dogs is one of the major clinical concerns, both in terms of mortality [1] and overall incidence [2]. For decades, human and veterinary medicine have been interconnected. This concept is defined as “one health–one medicine”, which has developed over the years, promoting the collaboration between physicians and veterinarians [3]. This is proven by the numerous comparative studies and animal models for human diseases. Many comparative studies have been performed on different tumor types affecting both humans and dogs, such as melanoma [4], osteosarcoma [5], and prostatic adenocarcinoma [6]. 

The approach for collecting scientifically-relevant papers for this review was based on a keyword-based overall search in the NCBI PubMed^®^ database of articles related to EMT and MET under physiological conditions and in different tumors, independently of the species. As a starting point and to have a wide overview of the topic, the article selection was based on the inclusion of both original research papers and reviews. The selection criteria were based on the previous knowledge of the authors about relevant literature and more recent papers on the topics presented in this review, while trying to avoid overlapping information and the inclusion of papers with non-univocal results. In addition, the main topics chosen were selected among those investigated by various research groups, in order to have different perspectives and evaluations. Preconceived exclusion criteria were not applied to any articles, thus research performed in a specific geographical area or institution was not prioritized. Particular attention was given to the recruitment of recent results available on these topics, reporting specific information on the cellular and molecular mechanisms supporting, or thought to support, EMT and MET processes and EMT/MET-related pathways. Then, the search focused on all available literature regarding EMT and MET in the canine species, which was gathered in the present review.

## 2. An Overview of Epithelial-to-Mesenchymal Transition and Mesenchymal-to-Epithelial Transition

### 2.1. The Metastatic Process Is a Complex and Challenging Multistep Event

The metastatic process is considered one of the hallmarks of cancer malignancy and an advanced stage [7]. The first steps of metastasis begin when malignant cells lose their connections with the surrounding cells and extracellular matrix (ECM), gaining motility and invasive features [8]. All these morpho-functional changes are possible in epithelial tumors because of a process called the epithelial-to-mesenchymal transition (EMT). This process is characterized by the downregulation of epithelial features and the prompt activation of the so-called “master gene regulators”, such that a mesenchymal phenotype is gradually developed [9]. Extensive studies have focused on this event, illustrating its role in various tumors, and proposed its use for prognostic, diagnostic, and even therapeutic approaches [10,11,12]. However, only a small percentage of circulating tumor cells (CTCs) survive this step and exit the circulation through extravasation. This latter process depends on many factors, including the blood flow [13] and endothelium remodeling [14]. Moreover, cell plasticity determines the interactions between CTCs and endothelium, allowing extravasation [15].

### 2.2. Epithelial-to-Mesenchymal Transition (EMT) 

Epithelial cells can acquire a mesenchymal phenotype as a consequence of the downregulation of epithelial cell hallmarks during the epithelial-to-mesenchymal transition (EMT) [16]. Micro-environmental signals triggers EMT. Cells in which the EMT process has just began are characterized by stable epithelial cell–cell junctions, apical–basal polarity, and interactions with the basement membrane. Changes in the gene expression and/or in the post-translational regulation result in the loss of these epithelial features and the acquisition of a mesenchymal phenotype. As a consequence, cells display a fibroblast-like morphology and increased cell motility [16,17]. The mesenchymal-to-epithelial transition (MET), i.e., the reverse process, can also occur afterwards. An interesting feature of EMT is that the cells can also reside in intermediate states and retain both epithelial and mesenchymal features. These intermediate states can be different, according to the biological context [16,18,19,20]. 

EMT is regulated by several transcription factors, as well as by other factors such as microRNAs, reactive oxygen species (ROS), and exosomes. Moreover, EMT is widely described in different phases of embryogenesis, fibrosis, and cancer development.

### 2.3. The Regulation of EMT

Multiple factors are involved in the regulation of EMT. For instance, the major transcription factors (TFs) of EMT include Zeb, TWIST, and SNAIL, and multiple networks involving TFs regulate EMT [21]. 

#### 2.3.1. Transcription Factors

Snail1 and Snail2 are induced by a vast plethora of molecules and pathways, such as TGF-β [22], Notch signaling [23,24], Wnt pathways [25,26], ROS [27], and hypoxic stress [28,29]. The short splicing variants of the transcription factors Singleminded-2 (SIM2s), ELF5, and ETS (E twenty-six)-domain transcription factor family member were also shown to directly bind the SNAIL2 promoter, to inhibit Snail2 transcription [30,31]. Interestingly, both SIM2s and ELF5 are essential for mammary gland ductal development and alveologenesis during pregnancy, and both are frequently lost during breast cancer development [30,31]. Mammary gland-specific knockout of either SIM2s or ELF5 hinders mammary gland development and also induces EMT-like changes in epithelial cells [30,31]. Since zinc fingers and other domains change in different Snail TFs [32], not all Snail family members trigger EMT with the same efficiency [33]. Snail family members not only repress the CDH-1 gene, as shown by Guaita and colleagues, but are capable of repressing other epithelial markers, such as claudins 1, 3, 4, 7, occludins, cytokeratins, and mucins [34].

Twist-related protein 1 (TWIST1) is a basic helix–loop–helix transcription factor encoded by the TWIST1 gene [35], having a basic domain interacting with the core E-box sequence “CANNTG”, a helix–loop–helix (HLH) domain that mediates homodimerization or dimerization with E12/E47, and a highly conserved C-terminal domain, called “Twist box” [36]. Twist is implicated in multiple epithelial cancers through its EMT promoting function and correlates with poor prognosis and invasion [14,37,38]. A role has also been described in embryogenesis, where it is defined as “neural crest specifier” for its role in neural crest formation, due to its EMT-promoting activity [39]. The interactions between Twist and other core EMT transcription factors are controlled by GSK3-mediated phosphorylation [40]. An interesting study by Lai and colleagues showed that EMT and cancer stemness properties can be obtained upon chronic treatment with TNF-α [40], a pro-inflammatory cytokine that activates Twist1 in a NF-κB-dependent fashion [41]. Twist represses E-cadherin, not only by binding CDH-1, but also by inducing Snail1 or Snail2, as Casas and colleagues showed in a study where knockdown of Snail2 resulted in no E-cadherin suppression by Twist1 [42]. 

The Zeb family members include ZEB1 and ZEB2, which contain multiple independent domains able to interact with other transcriptional regulators [43]. ZEB1 and ZEB2 activate N-cadherin and vimentin expression (markers of a mesenchymal-like phenotype) and repress E-cadherin binding to E-boxes of the CDH-1 promoter, as well as recruiting co-repressors [44]. In particular, E-cadherin repression is obtained by ZEB1 recruitment of a chromatin remodeling protein [45]. Phosphorylation of ZEB1 varies in different cell types [46], and both ZEB1- and ZEB2-repressing activities can be modulated by post-translational modifications, such as SUMOylation by Pc2 or acetylation [47]. Nicotinamide-adenine-dinucleotide-dependent histone deacetylase (SIRT1) is recruited by ZEB1 to repress the E-cadherin promoter and also induces ZEB and Snail factors, but not Twist [48]. Moreover, ZEB2 mRNA also functions as a competitive endogenous RNA (ceRNA) squelching other microRNAs (miRNAs) targeting other transcripts, thus activating their expression [49]. ZEB2 mRNA can also be controlled at protein level, for instance by YB-1, a protein associated with increased invasion in breast carcinomas [50]. Even steroid and growth hormones can upregulate ZEB in diabetic nephropathy and breast cancer [51,52]. In addition, the miR200 family can regulate ZEB1 and ZEB2 [53] through post-transcriptional control. Several other transcription factors have been described as involved in EMT regulation, including Goosecoid [54,55], LBX1 [56,57,58], FOXC2 [59,60,61], ETS-1 [62,63,64], and LEF-1 [65,66,67]. For instance, PRRX1 was found to induce EMT and worsen prognosis in colorectal, gastric, and breast cancers, whereas its silencing suppressed invasion, migration, cell proliferation, as well as EMT itself [68,69,70]. All these non-canonical EMT TFs have very specific direct roles in distinct tumors, but also interact with canonical EMT TFs such as Snail and ZEB, meaning that they may also indirectly affect EMT [71].

#### 2.3.2. MicroRNAs

miRNAs are non-coding RNA molecules with a 21–23 nucleotide length that regulate gene expression at post-transcriptional level [72,73,74]. MiRNAs regulate invasion and metastasis by targeting the transcripts of a large number of genes involved in EMT/MET. It was demonstrated that the loss of miR-200 correlates with the lack of E-cadherin expression in invasive breast cancer cell lines and breast tumor specimens, suggesting an in vivo role for the miR-200 family in EMT [53]. On the other hand, overexpression of individual miR-200 members or different clusters represses EMT, by directly targeting and downregulating ZEB1 and ZEB2. This results in enhanced E-cadherin expression and inhibition of murine mammary tumor cell migration and motility [75,76].

#### 2.3.3. Reactive Oxygen Species

Cells undergoing oxidative stress acquire adaptive mechanisms to counteract the potential toxic effects of elevated ROS, by promoting cell survival pathways and factors [77]. ROS activate numerous signaling pathways, including matrix metalloproteases (MMPs), integrins, EGF, EGFR, VEGF, TGF-β, HIF-1, HGF, NADPH oxidases, and p53 [78,79,80]. Interestingly, ROS play a key role in TGF-β1-induced EMT, primarily through MAPK activation and subsequently through ERK-dependent activation of the Smad pathway [81]. Another example of the role of ROS as second messengers for EMT was demonstrated by Bayurova and colleagues, showing that HIV-1 reverse transcriptase enhances tumor growth and metastasis formation via ROS-dependent upregulation of Twist [82].

#### 2.3.4. Exosomes and Microvesicles

A new metaphor for the “seed and soil” theory addresses extracellular vesicles as “fertilizers” of cancer cells (the “seed”) in their respective host organs (the “soil”) [83]. Exosomes are mediators of the crosstalk among metabolic organs, important factors for the organ specificity of metastasis [84,85,86] and EMT inducers [87,88,89]. Microvesicles are small membrane-enclosed structures thought to be shed from a variety of cell types and found in several body fluids [90,91]. They are morphologically distinct from exosomes and their dimensions are two-fold larger than exosomes. Other differences arise from their biogenesis and release [92]. The role of microvesicles has been described in various processes, including inflammation and coagulation [93], as well as in tumors [94]. Microvesicles can regulate MMP activity, favoring matrix degradation, a key step in the metastatic cascade that is often linked to EMT features [95,96]. Microvesicles can also be secreted and delivered to recipient cells, inducing them to undergo EMT [97]. Exosomes and microvesicles are classified as tumor-derived secreted factors (TDSFs), and their role in tumor signaling and metastasis is a topic of research, hopefully leading to new strategies for cancer diagnosis, cancer prognosis, and possibly cancer therapy in the future [98,99,100,101,102].

#### 2.3.5. Viruses

Increasing evidence has demonstrated that viruses can also trigger EMT, both in normal and neoplastic cells [103]. Among these, there are two very important viral groups: one is represented by the papillomaviruses, and the other by the coronaviruses. An increasing body of literature has demonstrated an association between high-risk papillomavirus infection and the onset of EMT, especially in cervical and oropharyngeal tumors [104]. E2, E6, and E7 papillomavirus oncoproteins have been further investigated in cervical cancers and it was demonstrated that these oncoproteins play a pivotal role in triggering EMT, favoring chemotherapy resistance and promoting a more aggressive biological behavior [105,106]. Interestingly, similar findings were also reported in veterinary medicine. An equine papillomavirus, i.e., *Equus caballus* papillomavirus type-2 (EcPV-2), also demonstrated this aspect in a species other than humans. Future studies focusing on papillomavirus-induced transformation can count on the horse as a promising spontaneous animal model. EcPV-2 infection in horses was reported to dysregulate RANKL and Wnt pathways [107], and induce EMT [108] in penile squamous cell carcinomas and EMT in a laryngeal squamous cell carcinoma [109].

Severe acute respiratory syndrome coronavirus-2 (SARS-Co2), the virus responsible for the pandemic that started in 2019, has been the focus of much research for the past 3 years. Interestingly, this virus is not only responsible for causing deaths due to its respiratory tropism and systemic dysregulation. Reports have described that SARS-CoV-2 infection in patients with cancer triggers the EMT phenomenon, thus favoring neoplastic malignant transformation [110,111,112]. In particular, a study by Lai and colleagues underlined the important oncogenic role of SARS-CoV-2 in triggering breast cancer metastasis through Snail upregulation [112]. Another study suggested that the SARS-CoV-2 M protein induces motility, proliferation, stemness, and in vivo metastasis of triple-negative breast cancer [111]. Despite studies reporting that SARS-CoV-2 could also infect felids [113], dogs [114], and many other domestic species [115], reports on dogs or other pets exhibiting a SARS-CoV-2-induced EMT process are still lacking.

### 2.4. EMT in Embryonic Life

EMT is an important mechanism in different phases of embryogenesis; in fact, most tissues undergo different cycles of EMT and its reverse program (MET) [16]. For instance, after gastrulation, and during the formation of the neural crest, EMT plays a pivotal role. Other tissues/organs, such as the kidneys, need EMT during embryogenesis, to become properly functional. Multiple rounds of EMT and MET need to take place [116]. Another example of physiologic EMT during embryogenesis is cardiac tissue development, in which some of the main signaling pathways, such as Notch, Bmp2, and Wnt/β-catenin, drive a primary EMT [117,118,119,120]. Cardiac valve formation follows a different path, thanks to a very similar process called endothelial-to-mesenchymal transition (EndMT), in which endothelial cells detach from vessels and form a mesenchymal phenotype [121]. Moreover, Ubil and colleagues showed the presence of the reverse process, called the mesenchymal-to-endothelial transition (MEndoT), during revascularization of the myocardium by cardiac fibroblast after ischemic cardiac injury [122].

### 2.5. EMT in Fibrosis

Fibrosis is a natural consequence of the repair of damaged tissue but can often be aberrant and uncontrolled, causing organ dysfunction [123]. During fibrosis, resident fibroblasts can acquire a smooth muscle cell-like phenotype, becoming myofibroblasts, and secrete excessive amounts of ECM [124]. Myofibroblasts are mainly involved in the physiologic remodeling process during healing [125,126]. One of the major drivers of fibrosis is TGF-β1, which has an important effect on myofibroblasts [127,128,129]. This mediator is also a critical regulator of EMT signaling and physiologic wound healing [129]. Common end-stage kidney fibrosis occurs in 30–40% of diabetic nephropathies [130] and it was postulated that EMT might play a role in this pathomechanism. EMT induces local fibroblasts to become myofibroblasts, as shown by Iwano and colleagues [131]. However, the origin of these myofibroblasts remains under debate [132,133,134]. They might arise from bone marrow, pericytes, renal epithelium, and vascular endothelium [135]. Nevertheless, other studies suggested that these different origins are not mutually exclusive and can be present at the same inflammation site [136]. 

Fibrosis is also one of the main events involved in hepatic cirrhosis [137]. During chronic liver diseases, the mechanisms driving or counteracting fibrosis are not balanced, leading to a persistent activation of hepatic myofibroblasts [138]. EMT also plays a role in this case and can lead to increased deposition of ECM in the hepatic parenchyma [139]. During this process, hepatocytes decrease the expression of E-cadherin and zonula occludens-1 (ZO-1) and replace them with expression of α-SMA, MMP-2, MMP-9, and vimentin [140]. In rats, TGF-β1 was shown to play a pivotal role in liver fibrogenesis [141], as it inhibits ECM degradation and promotes its deposition [142,143,144]. In addition, SMAD7 is involved in EMT-driven hepatic fibrosis. SMAD7 deletion promotes EMT, whereas overexpression protects against it [145,146], which is consistent with the process described in different tumors [147,148,149,150]. Improving the knowledge about EMT in hepatic fibrosis is of paramount importance for the development of possible new targeted therapies.

In veterinary medicine, a study by Aresu and colleagues used canine spontaneous tubulo-interstitial fibrosis to investigate EMT in tubular epithelial cells. The study tried to use a canine model to investigate whether tubular epithelial cells actively participate in the mechanism of renal fibrosis [151]. The study underlined that EMT of tubular epithelial cells might be one of the pathomechanisms involved during renal fibrosis in dogs, as reported in humans [151]. Another study in dogs, focusing on hepatic fibrosis, showed that the area and intensity of α-SMA staining in hepatic stellate cells (HSCs) strongly correlates with the increased fibrotic stage, giving a more contractile and profibrotic phenotype to these cells [152]. α-SMA is used in humans and rodents to confirm activation of HSCs and portal myofibroblasts in liver disease [153]. However, the reliability of this protein in dog hepatic fibrosis seemed not to be confirmed by other studies [152,154,155]. On the other hand, a study by Neumann and colleagues suggested that the plasma concentration of TGF-β1, a potent EMT-inducer, could be a good marker for hepatic fibrosis in dogs [156].

### 2.6. EMT in Cancer Metastasis

In 2008, Trimboli and colleagues were among the first to describe the occurrence of EMT in breast cancer, using the Rosa26LoxP reporter mouse model to genetically mark tumor epithelial and stromal cells independently and determine their fate during tumor progression. EMT resulted as only associated with myc-initiated breast tumors. This means that the occurrence of EMT to different degrees depends on the initiating oncogenes [157]. Various studies have shown quite different results, sometimes being contradictory, and the exact role of EMT during metastasis formation is under debate. Clusters of CTCs were found in the bloodstream of cancer patients in several studies, testifying to the presence of a collective migration in vivo [158,159]. Plakoglobin, an adherens junction protein, was found to be crucial for cluster formation, while its knockdown resulted in reduced metastatic spread [160]. Lecharpentier and colleague showed that the majority of isolated cells or clusters of CTCs in patients with advanced metastatic non-small cell lung cancer (NSCLC) show a dual epithelial–mesenchymal phenotype. This confirmed that EMT was an important process for invasion and metastasis in these patients [161]. In mouse, the role of Twist in lung cancer was investigated by Yang and colleague, showing that interfering with its expression through siRNA3 led to a drastic decrease of the number of metastases but did not prevent them [37]. In a study by Lu and colleague, it was shown that CTCs from human lobular breast cancer were predominantly epithelial, while those from HER2+ and triple-negative subtypes were mostly mesenchymal; this provided evidence of EMT in human breast cancer specimens [162], consistent with other studies in mice [163,164]. In another study, it was demonstrated that EMT inhibition by overexpressing miR-200 does not affect lung metastasis development, even though EMT plays a role in chemotherapy resistance [165]. In 2015, Zheng and colleague published a study defining EMT as “dispensable” for pancreatic cancer metastasis [166]. Aiello and colleague showed that EMT markers are expressed in micrometastases, whereas epithelial markers are re-expressed upon metastasis growth [167]. The same study also showed a link between myofibroblasts and metastasis dimension, as the number of associated myofibroblasts significantly increased with lesion size [167]. Tran and colleagues reported that Snail expression is sufficient to drive breast cancer cells into the circulation, but it must be downregulated once those cells reach the lung, to successfully colonize the pulmonary parenchyma [168].

### 2.7. Tumor Microenvironment (TME)

The tumor microenvironment (TME) consists of extracellular matrix components, especially collagen, fibronectin, hyaluronan, and laminin, as well as tumor cells, tumor stromal cells (including stromal fibroblasts), endothelial cells, and immune cells (microglia, macrophages, and lymphocytes) [169,170,171]. Modifications of the TME are essential for immune evasion during primary tumor growth [172,173,174,175]. Within the TME, different immunosuppressor cells can be found, including myeloid-derived suppressor cells (MDSCs) [176,177], cancer-associated fibroblasts (CAFs) [178,179], tumor-associated macrophages (TAMs) [180,181] and Treg lymphocytes [182,183], producing immunosuppressive mediators such as IL-10, TGF-β, VEGF, PGE2, and PD-L1 [184]. Tumor cells are able to produce TGF-β [185], which is a potent EMT driver. TGF-β also inhibits the immune response altering Fas ligand, IFN-γ, perforin, and other immune-related mediators [186]. A comprehensive review of EMT and the tumor microenvironment was provided by Romeo and colleagues [187].

### 2.8. Premetastatic Niches and EMT

Cancer cells need a favorable environment in order to metastasize [188]. Interestingly, primary tumors actively secrete factors to condition ECM and immune cell environment of a distant organ, thereby creating a supportive pre-metastatic niche, allowing the formation of metastasis [189]. Pre-metastatic niches can contain pro-tumor immune cells such as neutrophils [190], monocytes [191], macrophages [192], and bone marrow-derived cells (BMDCs) [193]. The latter are crucial for generating a suitable microenvironment for the primary tumor and the development of metastasis [194]. Tumor-derived exosomes (TDEs) can even recruit BMDCs through upregulation of pro-inflammatory molecules at pre-metastatic sites [195]. Hsu and colleagues showed that BMDCs cells can secrete extracellular vesicles containing miR-92a, specifically promoting lung cancer metastasis in the liver [196]. In addition, hypoxia can drive EMT [28,29,197] and promote pre-metastatic niche formation through HIFs and VEGFs [198]. Platelets were found to be crucial for the promotion of metastasis in some cancers, by improving survival of CTCs [199,200] and favoring the development of pre-metastatic niches [201]. In a study on a Lewis lung carcinoma spontaneous metastatic model, the knockout of the platelet ADP receptor (P2Y12) led to decreased lung fibronectin, a major component of pre-metastatic niches, resulting in decreased pulmonary metastasis [202]. As P2Y12 is a target for common anti-platelet drugs, it may be developed into a new target against metastasis [203].

### 2.9. EMT and Cancer Stem Cells (CSCs)

Stochastic genetic and/or epigenetic mutations in single cells were initially thought to be the cause of tumor heterogeneity. These changes were supposed to induce a clonal selection of cells with growth advantages. More recently, a hypothesis considering the involvement of cancer stem-like cells (CSCs) sustaining cancer progression has been formulated [204,205]. Subsequently, CSCs were found to be involved in drug resistance and metastatic dissemination [206,207,208,209]. Another hypothesis, which reconciles both abovementioned theories, suggests that cancer cell phenotypic plasticity, shifting between CSC and non-CSC states, is responsible for the evolution and maintenance of cancer [210] and that “stemness” in cancer cells could be a state, rather than an entity [211]. During the last decade, EMT has been identified as one of the key mechanisms that confers stem-cell properties. This notion came from a study on mammary gland and breast cancer cells, in which Snai2 and Sox9 interplay was found to determine a mammary stem cell fate [212]. Afterwards, the association between EMT and CSCs was recognized in many human carcinomas [213]. For instance, Rhim and colleagues demonstrated that in vivo pancreatic cancer cells that underwent partial EMT, expressing Zeb1 and E-cadherin, showed stem cell properties [164]. Another study showed that in vivo activation of slug and CD87 genes through their promoter demethylation was associated with EMT and cancer stem-cell features in lung cancer [214]. Interestingly, many EMT-associated miRNAs inhibit cancer cell stemness. However, miR-10b in breast cancer [215] and miR-1207-5p in colorectal cancer [216] block tumor-related suppressive signals, facilitating stemness properties [217]. 

The relationship between EMT and CSCs has also been investigated in veterinary medicine. Pang and colleagues isolated CSCs from a canine mammary carcinoma cell line (REM134) and showed that in vitro canine CSCs predominantly express mesenchymal markers and have enhanced invasive features. Subsequently, they showed that TGF-β can enhance CSCs features inducing EMT, thus increasing the ability to form tumor spheres [218]. Another in vitro study focusing on canine and human breast cancer cells showed that melatonin can modulate the expression of EMT-related proteins in breast CSCs, resulting in decreased tumor invasion [219].

### 2.10. EMT in Mammary Gland Carcinomas

Basal-like breast carcinomas, i.e., breast cancers showing triple-negative expression of estrogen receptor, progesterone receptor, and HER2 receptor, are among the most aggressive and deadly cancer subtypes, with a high metastatic ability associated with mesenchymal features [220,221]. One molecular pathway responsible for the mesenchymal phenotype in this cancer is the expression of SNAI2, driven by KRAS, a RAS oncogene family member [220]. Transcriptional profiling showed that EMT regulators were expressed in this carcinoma, but in a heterogenous fashion across the tumor [222]. Subsequently, a link between estrogen receptor (ER) silencing and EMT in human breast cancer cells was hypothesized. Findings have indicated that the loss of ERα probably results in an EMT phenomenon characterized by striking changes in the expression profile of specific matrix macromolecules [223,224]. High-grade carcinomas present higher numbers of cells positive for vimentin, nuclear β-catenin, and CD44, compared to low-grade carcinoma and benign lesions, suggesting that the breast cancer cell de-differentiation process could be related to EMT [220]. Basal-like carcinomas have more mesenchymal features compared to the luminal (A and B) and HER2-enriched counterpart and are correlated with more extended invasion [224,225]. An interesting series of effects is exerted by ET-1, one of the three isoforms of endothelin in mammary tumors, through its receptors ETAR (or ETA) and ETBR (or ETB). ET-1 is highly expressed in mammary tumors in humans and can modulate angiogenesis, invasion, apoptosis, and the metastatic potential via autocrine or paracrine action [226,227]. Chen and colleagues showed that TAMs induce the ET axis in endothelial and breast cancer cells through IL-8 and TNF-α secretion [228,229]. It is tempting to speculate that the role of ET-1 is linked to EMT, as it seems to be in other tumors such as chondrosarcoma [230]. EMT is widely involved in breast cancer metastasis [162,219,231], and research is focusing on its main pathways, to elucidate their role in these carcinomas and hypothesize future therapeutic approaches. The main targets are TGF-β [232]; silencing of CDH-1, which has shown controversial results [233,234]; Wnt/β-catenin pathway [235,236,237]; Notch [238]; TNF-α, through NF-κB-mediated transcriptional upregulation of Snail1 [41]; and miRNAs [237,239,240], especially miR-300-targeting Twist to inhibit EMT [241].

Canine mammary carcinomas are widely studied as a comparative model for humans [242]. Restucci and colleagues showed a correlation between ET-1 presence in canine mammary tumors (mostly G2- or G3-graded) and the malignancy of cancer, also suggesting a positive interaction between hypoxia and ET-1 expression [226]. Breast cancer incidence in humans is related to various environmental chemicals, including synthetic chemical bisphenol A (BPA) [243]. BPA is the main component used in the manufacturing of polycarbonate plastic, can be found in many common household products, and is present in air and drinking water [244,245]. An interesting study by Zhang and coll. showed that BPA stimulates EMT of estrogen negative breast cancer cells via FOXA1 signals [246]. As pet dogs fed with canned food showed high circulating BPA concentrations in serum, it would be intriguing to understand if this chemical product can induce EMT and impact canine mammary carcinoma incidence [247]. Another study investigating EMT in canine mammary carcinomas showed a positive correlation between E-cadherin+/vimentin+ cells and a higher tumor grade, and also reported some preliminary results on the possible role of SNAIL/SLUG transcription factors in the onset of metastasis, inducing EMT and subsequently MET. The study had some limitations, namely a restricted analysis of EMT markers and a small case number, but represents a promising starting point to focus future research on finding some meaningful predictive values of clinical outcome for canine carcinomas [248].

### 2.11. EMT in Prostatic Carcinomas

Prostatic tumors are a major cause of death in the human male population. Androgen deprivation therapy, one of the most common therapies against locally advanced and metastatic disease, is often ineffective [249]. The presence of an EMT-like phenotype has also been reported in prostatic tumors [250] and plays a role in both resistance to treatment and metastasis [251]. In prostate cancers, androgen signaling is dysregulated, allowing these hormones to suppress E-cadherin expression, and activate mesenchymal marker expression [252] and Snail [253]. Despite this, conflicting results have been published in recent years about the exact link between androgens and EMT [254,255,256,257,258]. Estrogens also play a role in prostate cancer through prostatic estrogen receptor alpha (ER-α) and beta (ER-β), whose expression patterns gradually differ during cancer progression [259]: ER-β inhibits the EMT process due to its inhibitory action on HIF-1α and Snail [260], while ER-β2 and ER-β5 variants can stabilize HIF-1α and favor hypoxic genes expression in prostate cancer [261]. EMT in prostatic cancer is promoted by both hypoxia and TGF-β signaling [262]. EGF and EGF receptor (EGFR) are aberrantly expressed in both androgen-independent and metastatic prostate cancers, with high EMT-related features, and are strongly associated with an aggressive phenotype, a poor clinical prognosis, a high Gleason score, and a reduced survival rate [263]. Moreover, EGF can induce Twist1 expression and prostate cancer cell invasion through a ROS/STAT3/HIF-1α signaling cascade [264]. Twist1 expression causes an increased expression of fibronectin and N-cadherin, and a concurrent E-cadherin decrease [265].

Evidence of EMT in canine prostatic cancers is available and comprises overexpression of vimentin [266], repression of E-cadherin expression [267], changes in β-catenin localization [268], loss of E-cadherin, and β-catenin translocation in prostatic metastases [269]. Taken together, the growing body of literature in canine prostatic cancer and EMT lays the basis for the possible use of a canine spontaneous animal model to further investigate treatment options or early prognostic markers.

### 2.12. EMT in Other Carcinomas

A high number of studies have investigated the role of EMT in the invasive tumor front (ITF) of oral squamous cell carcinoma (OSCC) and tongue squamous cell carcinoma (TSCC). In oral cancers, the morphological and functional features of the ITF are suggestive of the biological aggressiveness of oral cancers, showing cells with an increased aggressive metastatic potential [270]. The ITF shows increased cell invasion, motility, and several features of EMT, including vimentin expression [271], loss of E-cadherin [272], loss of claudins [273], and loss of laminin 5 [274]. In TSCC, an interesting link between EMT and tumor “budding” was studied, demonstrating that high-intensity tumor budding is associated with reduced E-cadherin expression and enhanced vimentin expression [275]. Tumor buddings show EMT features in esophageal adenocarcinomas, endometrial carcinomas, and colorectal carcinomas, and were shown to be a prognostic marker in colorectal and rectal carcinomas [276,277,278,279]. As tumors initiate inflammation, mediators and cytokines such as COX-2/PGE2, IL-6, ROS, RNS, miRNAs, and NF-κB are often involved in a series of cancers [280,281]. The role of COX-2/PGE2 was investigated in rectal cancers, showing that COX-2 expression was related to higher tumor stages. Interestingly, its expression was higher in metastatic lesions than in primary tumor lesions and related to lower E-cadherin expression, indicating that it probably induces an EMT-like phenotype [282]. This link between EMT and COX-2 could lead to future therapeutic approaches, as shown by the fact that the therapeutic role of COX-2 has been extensively studied, including in breast carcinomas and OSCC [272,283,284].

Concordant results have been achieved in veterinary medicine: in dogs, SCCs frequently have either oral or cutaneous origins, and a study by Nagamine and colleagues comparatively investigated EMT and the histological grading between these two SSCs. OSSCs resulted in a lower expression of β-catenin, desmoglein, and E-cadherin compared with the cutaneous ones. This lower expression of epithelial markers in the ITF and the presence of a few N-cadherin and vimentin immunolabelled cells was indicative of an EMT process in the tumor cells, predicting the biological behavior of canine SCCs. These results suggest that the investigation of the EMT process in canine oral and cutaneous squamous cell carcinomas may allow a more accurate prediction of their biological behavior [285]. Another more recent study underlined the importance of SLUG in promoting migration and invasion through EMT induction in a canine oral squamous cell carcinoma cell line [286].

The EMT process in canine melanomas has recently been described both in vitro and in vivo. A study from Schmid and colleagues described in vitro the EMT process in canine melanoma primary cell lines mostly obtained from amelanotic oral malignant melanomas [287]. On the other hand, an in vivo study confirmed the EMT process in canine melanomas characterized by expression of ZEB and Snail in tumor cells [288].

### 2.13. EMT as a Prognostic Marker

One of the aims of studying the EMT at different stages of cancer and metastasis is to find out whether it is possible to use it as a prognostic marker. Increasing evidence in many cancer types suggests that the presence of cells undergoing EMT could predict prognosis and biological behavior. In order to establish EMT-related prognostic markers, studies focused on different levels of EMT regulation are needed [289,290,291]. For instance, in human breast cancers, the loss of E-cadherin expression was successfully related to poor prognosis [292,293]. Martin and colleagues showed that the expression of the EMT master regulators Snail1, Slug, and Twist might be directly associated with higher mortality and metastasis in human breast cancer [294]. Similar results regarding EMT regulator expression were published for hepatocellular and ovarian carcinoma in humans [295,296,297,298]. Regarding the bladder, cancer can occur as muscle invasive bladder cancer (MIBC) or non-muscle invasive bladder cancer (NMIBC), the first having a worse prognosis and a five-year-survival rate of <50% [299]. In a study focusing on E-cadherin, vimentin, and Twist expression in bladder cancer, only vimentin seemed to be an independent predictor of cancer progression and reduced survival [300]. Cao and colleagues conducted a gene set variation analysis (GSVA), establishing a correlation between EMT and the transition from NMIBC to MIBC, and eventually developed an EMT signature that can be used as a negative prognostic marker [301]. Even though successful results have been published, EMT-related prognostic markers are not yet widely used for prognosis in clinical routine. One problem is that, in different locations of the same tumor, the expression of EMT markers can differ because of tumor heterogeneity. Moreover, these studies did not provide clear cut-offs for prognosis, which are instead present in other prognostic methods, such as the mitotic index, Ki67, Her2, and others [302,303,304]. One possible future application of these wide oncogenomic data sets is the creation of personalized medicine programs, allowing clinicians to obtain a cancer-specific and patient-specific prognosis [305].

In veterinary medicine, similarly to what was observed in human breast cancer, E-cadherin loss in canine mammary tumors has been related to a poor prognosis [306,307]. Another possible and interesting approach, and also more feasible in veterinary medicine, was described in a recent study. Furusawa and colleagues elegantly showed how performing immunocytochemistry (IHC) on cytology samples of canine and feline tumors allowed a fast assessment of tumor malignancy based on the IHC expression of EMT markers [308]. 

### 2.14. Mesenchymal-to-Epithelial Transition (MET)

Mesenchymal-to-epithelial transition (MET) is the reverse process of EMT, in which mesenchymal cells acquire an epithelial phenotype; it is observed under physiological conditions and in cancer [309]. In embryogenesis, many examples have been described [310]. MET is required for the formation of Langerhans islets during pancreatic development [311]. In heart embryogenesis, cardiac mesodermal cells acquire their mesenchymal phenotype through EMT at gastrulation [312], but then cardiac progenitors quickly become organized into a two-layered epithelium via MET. A secondary EMT occurs, and mesenchymal cells arising from this delamination form the endothelial cell lining of the heart through another MET, forming an endocardial tube surrounded by the myocardial epithelium. These tubes lead to the formation of the four compartments of the primordial heart. Another round of EMT, in this case more precisely EndMT, allows the formation of the endocardial cushion, i.e., the cells that later will assemble into the atrioventricular valvulo–septal complex [313]. In addition, nephrogenesis requires multiple rounds of EMT. In mammals, kidney arises from the metanephric mesenchyme and the ureteric bud. Reciprocal inductive interactions transform the ureteric bud into the renal collecting system, while the metanephric mesenchyme condenses and subsequently undergoes MET, to form to the nephrons [314]. The failure of cells to undergo MET can lead to the development of the pediatric kidney malignancy defined as Wilms’ tumor [315]. 

The role of EMT in cancer metastasis was apparently contradicted by findings revealing that distant metastases were largely composed of cells morphologically resembling primary tumor cells, instead of being composed of cells with a mesenchymal-like morphology. In some cases, metastatic lesions of carcinomas showed even higher E-cadherin levels than in the primary tumor [316,317,318]. To explain how this is possible, it could be argued that epithelial cancer cells are able to escape from the primary tumor site and reach distant sites for metastasis. This is in contrast with the strong evidence of a positive correlation between the loss of the epithelial phenotype and metastatic potential [319]. To further investigate this, Yates and colleagues cocultured human prostate carcinoma cells with hepatocytes, showing that this led to an increased expression of E-cadherin, and demonstrating that phenotypic plasticity can occur late in prostate cancer progression at the site of ectopic seeding [320]. One mechanism for the re-expression of E-cadherin in ectopic tissues was the loss of CDH-1 promoter demethylation, probably induced by the TME of the host organ [321]. These data and other studies provide a proof of principle that carcinoma cells may undergo MET, regaining E-cadherin, in response to the host organ microenvironment, to establish connections with the resident, non-neoplastic epithelial cells [322,323].

### 2.15. MET in Sarcomas

EMT in carcinomas is a widely studied mesenchymal state reversion involved in the pathogenesis of several carcinomas. Is it possible that, in sarcomas, a similar reversion occurs from a mesenchymal to an epithelial phenotype? Evidence of MET in sarcomas has been published in several studies, such as in synovial sarcomas [324], chondrosarcomas [325], epithelioid sarcomas [326], and leiomyosarcomas [327]. MET in sarcomas is characterized by an increased expression of classical epithelial markers, while tumor cells still predominantly express classical mesenchymal markers [328]. Epithelial markers in sarcomas show a higher expression and can be used as prognostic markers [329,330,331,332,333].

MET can be induced by several signaling pathways and cytokines, including c-MET [334]; platelet-derived growth factor receptor (PDGFR); fibroblast growth factor receptor (FGFR) through Fox-2 regulation [335]; TGF-β1, insulin-related growth factor 1 receptor (IGF1R), and regulatory kinases, such as phosphoinositide 3-kinase (PI3K), AKT, and mammalian target of rapamycin (mTOR) [309,322,324,336]. MicroRNAs are known to regulate EMT [337] and were also described as regulating MET [338,339,340,341]. MicroRNA cluster 302–367 was found to accelerate MET and induce somatic cell reprogramming [342]. One example is miR-147, found to primarily act by increasing E-cadherin expression and decreasing ZEB1 expression, which is a direct target. This results in the inhibition of cell motility and invasion in mouse cancer models [341]. ZEB1 is also targeted by miR150, in a similar pathway [340]. These data provide knowledge for the establishment of new prognostic markers, especially in tumors such as leiomyosarcoma or synovial sarcoma [343], in which the low incidence in humans does not help conduct research and statistical analysis.

Canine sarcomas, especially osteosarcomas (OSA), are in focus for comparative studies with humans, because they have a higher incidence, representing 9–15% of all cutaneous or subcutaneous tumors and 10–15% of all malignant tumors in dogs. It seems that 20% of these tumors originate in the bone and 80% is represented by soft tissue sarcomas (STS) [344,345]. Unfortunately, currently there are no studies focusing on the MET process in canine OSA. Another canine sarcoma that was widely characterized in vitro [346], and well-established in a murine xenotransplant model using canine cells [347,348] and in spontaneous tumors [349] is canine histiocytic sarcoma. This canine tumor represents a counterpart of a rare disease in humans, predominantly affecting the skin and soft tissues or occurring systemically [350,351]. This tumor has a very poor prognosis and an ideal treatment scheme is still lacking in both species [352]. For this reason, canine histiocytic sarcoma is a very interesting translational animal model, especially due to the relatively high prevalence of this tumor in dogs [353]. Recently, MET has been described in a histiocytic sarcoma cell line (DH82) in vitro, characterized by increased expression of epithelial markers at protein level, namely E-cadherin and cytokeratin 8, and activation of pathways involved in MET at transcriptome level. These changes were associated with decreased cell motility and invasion on matrigel, which were interpreted as a decreased aggressive biological behavior. Based on the in vitro results, it was postulated that MET in canine histiocytic sarcoma could be used as a favorable prognostic factor [346]. The authors would like to point out that histiocytic sarcoma is discussed in this section because, despite a non-mesenchymal cell origin, the MET in these cells results in decreased tumor invasion, similarly to human sarcomas undergoing MET. Investigations of MET in sarcomas in canine tumors remains an open field in veterinary medicine. To date, only data from preliminary studies on canine perivascular wall tumors are available [354].

### 2.16. Therapeutic Approaches for EMT and MET

The immune response is one of the key targets studied in order to obtain tumor regression. Research has focused on the transfer of naturally occurring or gene-engineered T cells, called adoptive immunotherapy [355], and on re-activating these T cells through the action of immune checkpoint inhibitors [356]. The most interesting results in various cancers come from studies of three antibodies: anti-programmed cell death protein 1 (anti-PD-1), anti-programmed cell death protein ligand 1 (anti-PD-L1), and anti-cytotoxic T-lymphocyte antigen 4 (anti-CTLA-4) [183,357,358,359,360]. A large number of patients show acquired or intrinsic resistance. The latter is possible because the genomic and epigenomic instability of transformed cells allows certain resistant phenotypes to be naturally selected [361]. Acquired resistance occurs when single tumor cells are able to survive or escape immunity. One perfect example is provided by the exploitation of immune homeostasis mediators by cancer cells, such as PD-1 in response to IFN-γ [362]. A study by Mak and colleagues showed a positive correlation between the EMT signature and the high expression of several immune checkpoints, including PD-1, PD-L1, PD-L2, B7-H3, OX40, OX40L, CD137, TIM3, LAG3, and CTLA4 [363]. EMT is also responsible for drug resistance to anti-cancer chemotherapies [213,364]. For instance, in preoperative chemotherapy-treated patients with esophageal cancer, both increased SNAIL1 and decreased E-cadherin expression were predictive of poor chemotherapy response and lower overall survival [365]. Can EMT be targeted to favor tumor regression in carcinomas? The microRNA-200 family is proven to be a potent repressor of EMT [53], and the upregulation of some of its members can increase sensitivity to chemotherapy in both chemoresistant prostate carcinoma and pancreatic cancer cells [366,367,368].

An interesting new approach in cancer therapy research is the use of nanocarriers, usually made of noble materials. One of the advantages of these nanostructures is that they overcome most of the obstacles found with traditional drugs, such as the lack of specificity for cancer cells [369]. Nanocarriers do not cause cytotoxicity [370] and have intrinsic properties, so that the use of unmodified nanoparticles exerts different effects [371]. Arvizo and colleagues showed that administration of unmodified gold nanoparticles (AuNPs) induced a reversion of EMT in ovarian cancer models, inducing a higher expression of E-cadherin, and lower expression of vimentin and Snail [372]. Another study of a similar cancer model showed that AuNPs also induced higher sensitivity to cisplatin [373]. Similar studies have also been published for pancreatic cancer [374]; for melanoma, where AuNPs also reduced metastasis [375]; and for others [376,377]. Some studies focused on the delivery of small molecules such as short interfering RNAs or miRNAs, for instance those of the miR-200 family, to reduce metastasis and tumor growth [378,379]. In the future, one possible application of this knowledge would be directly targeting cancer cells undergoing EMT, to block their progression towards increased proliferation, acquisition of stem-like property, increased metastatic potential, and chemoresistance.

Cytotoxic T lymphocytes (CTLs) mainly use the perforin/granzyme pathway to destroy target cells, including cancer cells [380]. Interestingly, it was shown that cells with experimentally-induced high expression of Brachyury, an EMT inducer, had decreased susceptibility to lymphocyte-mediated killing [381]. Another link between EMT and immune escape was hypothesized by Akalay and colleagues, in a study where MCF-7 human mammary carcinoma cells underwent EMT and exhibited reduced susceptibility to CTL-mediated lysis. This was possible through stable expression of SNAIL or after prolonged exposure to TNF-α [382]. Interestingly curcumin, a phytochemical derived from *Curcuma longa*, showed EMT repression in the same cancer cells [383]. Nonetheless, no therapeutic strategy to date has specifically targeted EMT to obtain an increase of CTL activity.

Vimentin is highly expressed in carcinoma cells that undergo EMT, and new drugs targeting vimentin might lead to improvements in cancer therapy. Withaferin-A (WFA) is a steroidal lactone derived from *Withania somnifera*, a medicinal plant commonly used in India, and showed anti-cancer properties, including the repression of tumor growth and tumor-associated angiogenesis [384,385]. By focusing on the exact mechanisms of WFA, it was discovered that it acts through the degradation of vimentin [386]. Another study also showed that knockdown of vimentin in cancer cells makes them less sensitive to WFA [387]. The latter results were obtained in sarcoma cells and are promising for the future development of anti-vimentin therapies in soft tissue sarcomas (STS) [387]. Another promising approach in veterinary medicine was described in vitro by Armando and colleagues, infecting canine histiocytic sarcoma cells with an oncolytic virus, namely canine distemper virus-Onderstepoort (CDV-Ond). This induced a perinuclear accumulation of vimentin that in turn resulted in decreased cell motility and invasion of matrigel by tumor cells [346]. An interesting therapeutic approach for canine mammary tumors was reported by Ren and colleagues, showing that miR-124 regulates canine mammary carcinoma growth and EMT in vitro by targeting CDH2, and thus suggesting a potential therapeutic strategy against canine mammary carcinoma [388].

## 3. Conclusions

This review provides an overview of knowledge about the EMT and MET processes in both human and canine species. However, to answer the question “how far have we come with EMT and MET knowledge in canine tumors?”, it is evident that human medicine is advancing at a different pace. All relevant findings in veterinary medicine fields are summarized in Table 1. While a large amount of information is available for humans, veterinary medicine is not yet at the same level. This situation should not discourage, but rather motivate, veterinary medicine researchers to apply the knowledge from the human counterparts to research in pets. By merging the knowledge from these two disciplines, better and faster results can be reached, thus improving human and canine health.

## Figures and Tables

**Table 1:** Main results of epithelial-to-mesenchymal transition (EMT) and mesenchymal-to-epithelial transition (MET) reported in the canine species.

Type of Disease	Type of Process	Main Results	Study Conditions	Ref.
renal fibrosis	EMT	proved EMT of tubular epithelial cells during renal fibrosis	in vivo	[151]
hepatic fibrosis	EMT	α-SMA staining in hepatic stellate cells strongly correlates with an increased fibrotic stage	in vivo	[152], but not confirmed by [154,155]
hepatic fibrosis	EMT	TGF-β1 plasma concentration could be a good marker for hepatic fibrosis	in vivo	[156]
mammary carcinoma	EMT	TGF-β induces EMT in cancer stem cells, enhancing tumor sphere formation	in vitro	[219]
mammary carcinoma	EMT	melatonin modulates EMT-related protein expression in cancer stem cells resulting in a decreased tumor invasion	in vitro	[220]
mammary carcinoma	EMT	correlation between ET-1 and the malignancy of the neoplasm, suggesting a positive interaction between hypoxia and ET-1 expression	in vivo	[227]
mammary carcinoma	EMT	positive correlation between E-cadherin+/vimentin+ cells and higher tumor grade	in vivo	[250]
mammary carcinoma	EMT	E-cadherin loss is related to a poor prognosis	in vivo	[308,309]
mammary carcinoma	EMT	miR-124 regulates EMT by targeting the CDH2 gene	in vitro	[388]
prostatic carcinoma	EMT	EMT in canine prostate gland carcinoma features vimentin over-expression, E-cadherin loss and β-catenin nuclear translocation	in vivo	[268,269,270,271]
oral and cutaneous squamous cell carcinoma	EMT	oral squamous cell carcinoma undergoes EMT showing an N-cadherin and vimentin expression, and a lower expression of β-catenin, desmoglein, and E-cadherin compared to cutaneous carcinoma	in vivo	[287]
oral squamous cell carcinoma	EMT	importance of SLUG in promoting migration and invasion through EMT induction in a canine oral squamous cell carcinoma cell line	in vitro	[288]
melanoma	EMT	the EMT process is characterized by secretion of biologically-active MMP2	in vitro	[289]
melanoma	EMT	the EMT process is characterized by ZEB and Snail expression in tumor cells	in vivo	[290]
histiocytic sarcoma	MET	increased expression of epithelial markers (E-cadherin and cytokeratin 8) associated with a decreased aggressive biological behavior of tumor cells	in vitro	[348]

α-SMA: alpha smooth muscle actin; TGF-β1: tumor growth factor beta 1; ET-1: endothelin 1; E-cadherin: epithelial cadherin; miR: microRNA; CDH2: cadherin 2; N-cadherin: neural cadherin; SLUG: Zinc finger protein SNAI2; MMP2: matrix metalloprotease 2; ZEB: zinc finger E-box binding homeobox; Snail: Zinc finger protein SNAI1.

## Data Availability

Not applicable.
